# LncRNAH19 acts as a ceRNA of let-7 g to facilitate endothelial-to-mesenchymal transition in hypoxic pulmonary hypertension via regulating TGF-β signalling pathway

**DOI:** 10.1186/s12931-024-02895-y

**Published:** 2024-07-10

**Authors:** Xin Yu, Jiabing Huang, Xu Liu, Juan Li, Miao Yu, Minghui Li, Yuliang Xie, Ye Li, Junyu Qiu, Zhou Xu, Tiantian Zhu, Weifang Zhang

**Affiliations:** 1 Department of Pharmacy, The Second Affiliated Hospital of Nanchang University, No. 1 Minde Road, Nanchang, 330006 Jiangxi P.R. China; 2 Department of Pharmacology, School of Pharmaceutical Science, Nanchang University, Nanchang, 330006 Jiangxi P.R. China; 3 Department of Cardiology, The Second Affiliated Hospital of Nanchang University, Nanchang, 330006 Jiangxi P.R. China; 4 College of Pharmacy, Xinxiang Medical University, No. 601 Jin-sui Road, Xinxiang, 453003 Henan P.R. China; 5 Henan International Joint Laboratory of Cardiovascular Remodelling and Drug Intervention, Xinxiang, 453003 Henan P.R. China; 6 Department of Pharmacy, The First Affiliated Hospital of Xinxiang Medical University, Xinxiang, 453003 Henan P.R. China; 7 The First Clinical Medical College, Jiangxi Medical College, Nanchang University, Nanchang, Jiangxi China; 8 Queen Mary School, Medical Department, Nanchang University, Nanchang, 330031 China; 9 The Second Clinical Medical College, Jiangxi Medical College, Nanchang University, Nanchang, Jiangxi China

**Keywords:** Hypoxic pulmonary hypertension, lncRNA-H19, Endothelial-to-mesenchymal transition, microRNA-let-7 g, TGFβR1

## Abstract

**Background:**

Hypoxic pulmonary hypertension (HPH) is a challenging lung arterial disorder with remarkably high incidence and mortality rates, and the efficiency of current HPH treatment strategies is unsatisfactory. Endothelial-to-mesenchymal transition (EndMT) in the pulmonary artery plays a crucial role in HPH. Previous studies have shown that lncRNA-H19 (H19) is involved in many cardiovascular diseases by regulating cell proliferation and differentiation but the role of H19 in EndMT in HPH has not been defined.

**Methods:**

In this research, the expression of H19 was investigated in PAH human patients and rat models. Then, we established a hypoxia-induced HPH rat model to evaluate H19 function in HPH by Echocardiography and hemodynamic measurements. Moreover, luciferase reporter gene detection, and western blotting were used to explore the mechanism of H19.

**Results:**

Here, we first found that the expression of H19 was significantly increased in the endodermis of pulmonary arteries and that H19 deficiency obviously ameliorated pulmonary vascular remodelling and right heart failure in HPH rats, and these effects were associated with inhibition of EndMT. Moreover, an analysis of luciferase activity indicated that microRNA-let-7 g (let-7 g) was a direct target of H19. H19 deficiency or let-7 g overexpression can markedly downregulate the expression of TGFβR1, a novel target gene of let-7 g. Furthermore, inhibition of TGFβR1 induced similar effects to H19 deficiency.

**Conclusions:**

In summary, our findings demonstrate that the H19/let-7 g/TGFβR1 axis is crucial in the pathogenesis of HPH by stimulating EndMT. Our study may provide new ideas for further research on HPH therapy in the near future.

**Supplementary Information:**

The online version contains supplementary material available at 10.1186/s12931-024-02895-y.

## Introduction

Pulmonary hypertension (PH) is a refractory pulmonary vascular remodelling disease [1]. Hypoxia has been identified as a high-risk factor inducing the development of PH [2]. Clinically, hypoxic pulmonary hypertension (HPH) has been classified as the third category of this disease, which is common in individuals with chronic lung disease or living at high altitudes [3]. Although a growing armamentarium of available therapeutics, such as vasodilators, anticoagulants and diuretics, has significantly improved the management of the disease in the past decade, few cases of HPH have been eradicated [4, 5]. The 3-year survival rate of HPH patients is significantly worse than that of patients with other diseases [6]. 

Pulmonary artery remodelling, which results from an excessive increase in pulmonary artery smooth muscle cells (PASMCs), is a key event of HPH, leading to angio-obliterative vascular structural changes and excessive vasoconstriction [7, 8]. The increased in PASMCs can be derived from resident PASMCs itself, epithelial cells, fibroblasts and pericytes [9]. Recently, pulmonary artery endothelial cells (PAECs) were shown to contribute to vascular remodelling in HPH though their transformation into mesenchymal or SM-like phenotype cells, which called endothelial-to-mesenchymal transition (EndMT), that then migrate into their underlying tissues [10, 11]. Other studies also shown that inhibition of EndMT can attenuate pulmonary vascular remodelling and reduce pulmonary artery pressure [12]. Therefore, EndMT would be a promising therapeutic target for HPH treatment.

Long noncoding RNAs (lncRNAs), a class of noncoding RNAs containing more than 200 nucleotides, participate in various biological activities by functioning as competing endogenous RNAs (ceRNAs) that compete for microRNA (miRNA) binding, thereby controlling the stability or translation of mRNAs targeted by miRNAs and altering their response to various stimuli at the transcriptional and posttranscriptional levels [13]. LncRNA-H19 (H19) is an imprinted gene located on chromosome 11 that is barely detectable in healthy adult animals but prominently expressed in endothelial cells after blood vessel injury [14, 15]. Previous studies have shown that H19 is closely related to many cardiovascular diseases, such as myocardial ischaemia, heart failure and atherosclerosis [16, 17]. Notably, the latest studies have proven that the H19 level was significantly increased in the blood of patients with end-stage idiopathic PAH and positively correlated with the degree of right ventricular hypertrophy [18]. However, whether H19 is involved in the pathological progression of HPH and its potential function remain largely unclear. Thus, we wanted to explore whether H19 was necessary for EndMT in HPH.

In the present study, we investigated the role of H19 in pulmonary artery remodelling and EndMT in H19-deficient rat HPH model. We further explored the underling molecular mechanisms of H19 function during the EndMT in both primary rat PAECs and human PAECs under hypoxic conditions.

## Materials and methods

### Data collection and analysis of differentially expressed genes

The method we used is similar to that reported in an earlier study we published [19]. Briefly, the gene expression profiling datasets GSE24988 [20], GSE113439 [21] and GSE117261 [22] are based on GPL6244 ([HuGene-1_0-st] Affymetrix Human Gene 1.0 ST Array [transcript (gene) version]) were downloaded from the GEO database (https://www.ncbi.nlm.nih.gov/geo/). The GSE24988 dataset contained 62 PH and 22 normal lung tissues. The GSE113439 dataset contained 15 PH and 11 normal lung tissues. The GSE117261 dataset contained 58 PH and 25 normal lung tissues. The 3 datasets were merged and normalized using the “sva” R package. We identified differentially expressed genes (DEGs) using the “limma” package in R. Values with *P* < 0.05 and |log2Fold change (logFC)| >0.5 were considered statistically significant.

### HPH rat model

H19-deficient rats (H19−/−) on a Sprague Dawley background were purchased from Cyagen Biosciences Inc. and bred in our animal feeding room under controlled conditions (12 h light/dark cycle, 23 ± 2 °C). After that, the tails of the offspring rats were isolated and genotyped by PCR (Figure S1A-B). Eight-week-old male homozygous rats and their wild-type (WT) littermates were used in the experiment.

To induce HPH, rats were placed in an atmospheric hypoxia incubator (Changjin, Changsha) at 10% O_2_, and the control rats were maintained in normoxic conditions for 4 weeks. All experimental procedures involving rats were carried out during the study following the principles approved by the University of Xinxiang Animal Care and Use Committee.

### Echocardiographic assessment

After 4 weeks of hypoxia treatment, the rats were anaesthetized with isoflurane (2%) and imaged with a VEVO 2100 imaging system (Visual Sonics, Ontario, Canada) equipped with a 30 MHz probe. Stable images were obtained in M and Doppler modes, and the acceleration time (PAAT) and ejection time (PAET) of the pulmonary artery and tricuspid annular plane systolic excursion (TAPSE) were measured.

### Right ventricular systolic pressure (RVSP) and mean pulmonary artery pressure (mPAP) measurement

The rats were anaesthetized, and their right external jugular veins were stripped and slit. Then, the PE catheter filled with heparin saline and connected with a pressure transducer (TaiMeng, Chengdu, China) was slowly inserted into the blood vessel from the incision. The right ventricular end systolic pressure (RVSP) and mean pulmonary artery pressure (mPAP) was recorded in real time after the pressure waveform stabilized.

### Sampling

After RVSP measurement, all rats were sacrificed under anaesthesia, and the heart samples were removed. The ratio of right ventricle to left ventricle plus ventricular septum (RV/LV + S) and right ventricular weight to tibial length (RV/TL) were used as indices of right ventricular hypertrophy. Meanwhile, the lung tissue and secondary branches of the pulmonary artery of all rats were collected. A portion of the lung tissue samples was stored at -80 ℃, and the other lung tissue was soaked in 4% paraformaldehyde solution. The pulmonary arteries were kept in precooled electron microscope fixative (2.5% glutaraldehyde) at 4 ℃ overnight.

### Transmission electronic microscope (TEM)

The pulmonary arteries immersed in 2.5% glutaraldehyde were trimmed and fixed in 1% osmic acid fixative for 3 h. After dehydration with gradient alcohol and soaking in Embed 812 (14,120, SPI, USA) overnight, all pulmonary arteries were baked and solidified in an oven at 60 ℃ for 48 h and subsequently cut into 70-nm slices. Afterwards, the slices were stained with 3% uranium acetate lead citrate and observed and photographed by transmission electron microscopy (Phillips, Netherlands).

### Morphological staining

Lung tissues were fixed with 4% paraformaldehyde for 16 h, dehydrated with gradient alcohol, and subsequently embedded in paraffin. Then, the tissues were sliced into 4-μm sections, stained with haematoxylin and eosin stain (HE), Masson or van Gieson (VG) as per the standardized protocols, and observed by light microscopy (Olympus, Japan).

### In situ hybridization (ISH)

An in situ hybridization (ISH) assay was conducted using an RNA ISH Kit (GDP1061, Servicebio, Wuhan, China) according to the manufacturer’s protocol. Briefly, paraffinized lung tissue sections were exposed to mRNA fragments using citric acid and Protease K, endogenous peroxidase activity was blocked with 3% H_2_O_2_, and the sections were reacted with prehybridization solution at 37 ℃ for 1 h. Then, the slices were incubated in H19 probe hybridization solution at 42°C overnight. After rinsing with SSC three times, the slices were incubated in hybridization solution with a secondary probe and blocked with 3% FBS for 30 min. Next, the sections were reacted with anti-DIG-HRP for 50 min, washed with PBS, and developed with DAB for 10 min. Finally, the sections were counterstained with haematoxylin for 3 min and observed under an optical microscope (Olympus, Japan). The sequence of the H19 probe was 5’-GGGCTAGAGGCTTGGCTCCAGGATGATGT (ttt CATCATCAT ACATCATCAT) 30 − 3’, and the sequence of the secondary probe was 5’-DIG-tt-ATGATGATGT ATGATGATGT-3’.

### Hypoxia-induced HPH in PAECs

Primary rat pulmonary artery endothelial cells (RPAECs) were isolated from pulmonary arteries by the collagenase digestion method and then enriched by magnetic sorting. Briefly, male Sprague‒Dawley rats (200–350 g) were sacrificed, and their pulmonary arteries were excised. The pulmonary arteries were cut thoroughly and digested with collagenase I for 1 h. After 200 mesh cell sieve filtration, CD31-FITC was added to the cell suspension, and RPAECs were enriched by magnetic sorting. The cells were maintained in EBM-2 (Lonza) supplemented with 10% fetal bovine serum (FBS) (HyClone) at 37 °C in the presence of 5% CO_2_. Primary cells were allowed to grow and were passaged at confluency by trypsin digestion into culture flasks. RPAECs were characterized by indirect immunofluorescence using an antibody specific to rat CD31 and α-SMA (Figure S2). We carried out follow-up experiments with cells within 5 generations.

human pulmonary artery endothelial cells (HPAECs) purchased from XinYu Biotechnology Inc. (XY-h443, Shanghai, China) were grown in special culture medium for endothelial cells (CP0028, XinYu) containing 10% FBS (WGG8001-100, Servicebio, Wuhan, China) at 37 °C.

To establish an HPH model in PAECs, RPAECs or HPAECs that reached 50% confluence were starved with medium with a serum concentration of 0.02% for 12 h and then cultured in an anoxic incubator (3% O_2_) for 48 h.

### Cell transfection

Small interfering RNA (siRNA) oligonucleotides for H19 (H19-si), TGFβR1 (TGFβR1-si), siRNA negative control (NC-si), miR-let-7 g-5p mimic and mimic negative control (mimic-NC), miR-let-7 g-5p inhibitor and inhibitor negative control (inhibitor-NC) were generated by RiboBio Biotechnology Inc. (Guangzhou, China). Cell transfection was performed in PAECs via a riboFECT CP Transfection Kit (C10511-05, RiboBio) in accordance with the manufacturer’s protocol. After 12 h, the transfected cells were collected and subsequently treated with hypoxia.

### Immunofluorescence

After transfection and chronic hypoxia treatment, the HPAECs cultured on sterilized coverslips were fixed with 4% paraformaldehyde for 20 min. Then, the cells and lung sections were reacted with 0.5% Triton X-100 and blocked with 3% FBS (WGG8001-100, Servicebio) at room temperature for 1 h. Afterwards, the cells and sections were incubated with primary antibodies against CD31 (1:1,000 dilution; cat. no. ab9498; Abcam) or α-SMA (1:50 dilution; cat. no. ab150301; Abcam) overnight at 4 °C and subsequently counterstained with the FITC/Cy3-conjugated secondary antibody (1:400 dilution, A11008, affinity). After rinsing with PBS three times, the coverslips and tissue sections were stained with DAPI for 5 min at room temperature. The results were imaged using a fluorescence microscope (Olympus, Japan).

### Dual luciferase reporter assay

The sequences of H19 containing wild-type (WT) or mutant (MUT) let-7 g-5p binding sites were generated and cloned into the pmiR-RB-Report™ luciferase reporter vector (Ribobio), generating corresponding constructs H19-WT and H19-MUT. Similarly, the 3′UTR of TGFβR1 containing let-7 g-5p binding sites or its corresponding mutant was used to generate TGFβR1-WT and TGFβR1-MUT on the basis of the pmiR-RB-Report™ luciferase reporter vector. For the dual luciferase reporter assay, HEK293T cells were cotransfected with let-7 g-5p mimic or mimic-NC and constructed vectors by lipofectamine™ 3000. After 48 h, luciferase activity analysis was performed using an ONE-Glo™ EX Luciferase Assay System (E8110, Promega, USA), with Renilla luciferase activity as a control.

### qRT‒PCR

Total RNA from pulmonary arteries or PACEs was extracted by using TRIzol (Invitrogen, ON, Canada). Then, the cDNA for PCR was produced using 2 μg total RNA and KEIris RT mix with dsDNase (All-in-One) (Codonx, Beijing, China). Next, RNA expression levels were evaluated by using TB Green^®^ Premix Ex Taq™ II Kit (Takara, Japan) on an Azure CieloTM real-time PCR system (Azure Biosystems, USA). The specific primers were designed using primer 3 and are listed as follows:

h-H19-Forwards: 5’-CGTGACAAGCAGGACATGACA-3’.

h-H19-Reverse: 5’-CCATAGTGTGCCGACTCCG-3’.

r-H19-Forwards: 5’-CTAAGTCGATTGCACTGGTTTGG-3’.

r-H19-Reverse: 5’-ACACCCAGTTGCCCTCAGAC-3’.

### Western blot

The total protein content was extracted by precooled lysis buffer and quantified by bicinchoninic acid (BCA) protein assay kit (WB6501, New Cell & Molecular Biotech LTD, Suzhou, China). Subsequently, equal samples containing 30 μg proteins were electrophoretically separated via 10% SDS‒PAGE and then blotted onto polyvinylide fluoride (PVDF) membranes. The proteins were blocked with blocking buffer (P30500, New Cell & Molecular Biotech) for 15 min and then incubated overnight at 4 °C with primary antibodies against CD31 (AF6191, 1:1000), α-SMA (AF1032, 1:1000), Vimentin (AF7013, 1:1000), TGFβR1 (AF5347, 1:1000) and β-actin (AF7018, 1:10000). The membranes were incubated in the HRP-linked anti-rabbit IgG secondary antibody (S0001, 1:6000) for 1 h. All the above mentioned antibodies were purchased from Affinity Biosciences LTD (CA, USA). Finally, the membrane was immunostained with a Bio-Rad image analysis system (Bio-Rad Inc., CA, USA) using an ECL kit (P2300, New Cell & Molecular Biotech).

### Statistical analysis

The data are presented as the means ± SEMs, and significant differences among groups were analysed using the unpaired t test (two groups) or one-way ANOVA test (more than two groups) in Statistical Product and Service Solutions (SPSS) 19.0 (Systat Software, San Jose, CA, USA). *P* < 0.05 was deemed statistically significant.

## Results

### H19 was increased in the lung tissue of PH patients according to DEG analysis

**Fig. 1:**
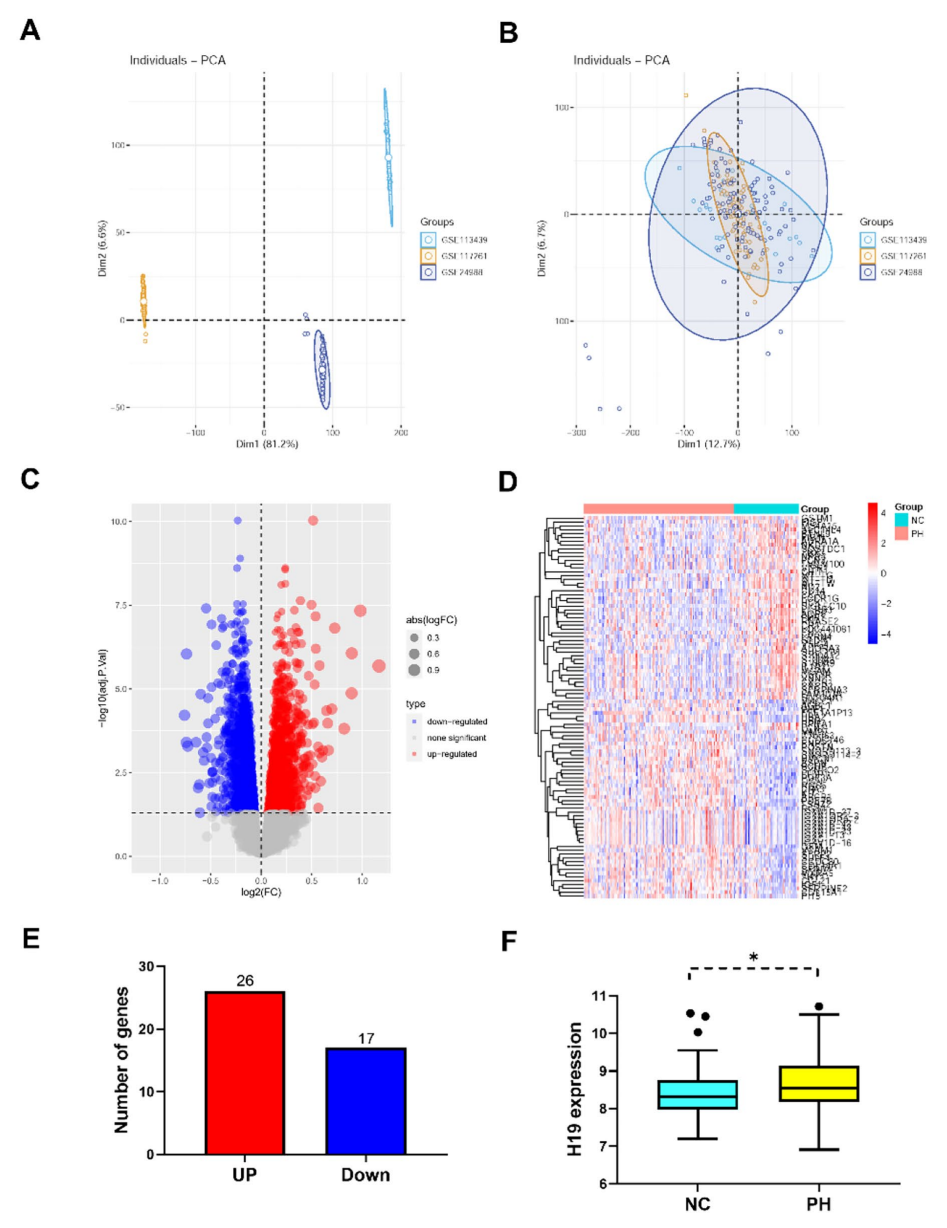
H19 was increased in lung tissue of PH patients through differential expressed gene (DEG) analysis. (**A**) PCA before combined; (**B**) PCA after combined; (**C**) Volcano map of DEGs. Magenta dots represent genes with |logFC| > 0.5 and adj.Pval < 0.05. The blue nodes on the left represent downregulated DEGs and red nodes on the right represent upregulated DEGs; the gray nodes represent genes with *p*-value > 0.05; (**D**) Heatmap of 43 DEGs. The diagram presents the result of a two-way hierarchical clustering of all the DEGs and samples. Each row in the heatmap represents a sample, and each column represents gene. The color scale at the right of the heatmap represents the raw Z-score ranging from blue (low expression) to red (high expression); (**E**) H19 expression in lung tissue of PH patients through DEG analysis

We performed DEG analysis which method similar to that reported in an earlier study we published. In this paper, we analysed 135 PH and 58 normal samples of lung tissue after combining the GSE24988, GSE113439 and GSE117261 datasets (Fig. 1A-B). The limma program was used to compare the DEGs between the two groups and found 43 DEGs. These DEGs may be able to differentiate between PH and normal patients, according to heatmaps created using hierarchical cluster analysis (Fig. 1C). We used R to analyze the GSE24988, GSE113439, and GSE117261 datasets, and volcano plots were used to show the differences between PH and normal tissues (Fig. 1D). In the two sets of differentially expressed genes, we discovered 26 upregulated and 17 downregulated genes (Fig. 1E). According to the GEO database, we observed that the expression of H19 was noticeably higher in the lung tissue of PH patients (Fig. 1F).

### H19 was increased in HPH rats and hypoxia-treated PAECs

**Fig. 2:**
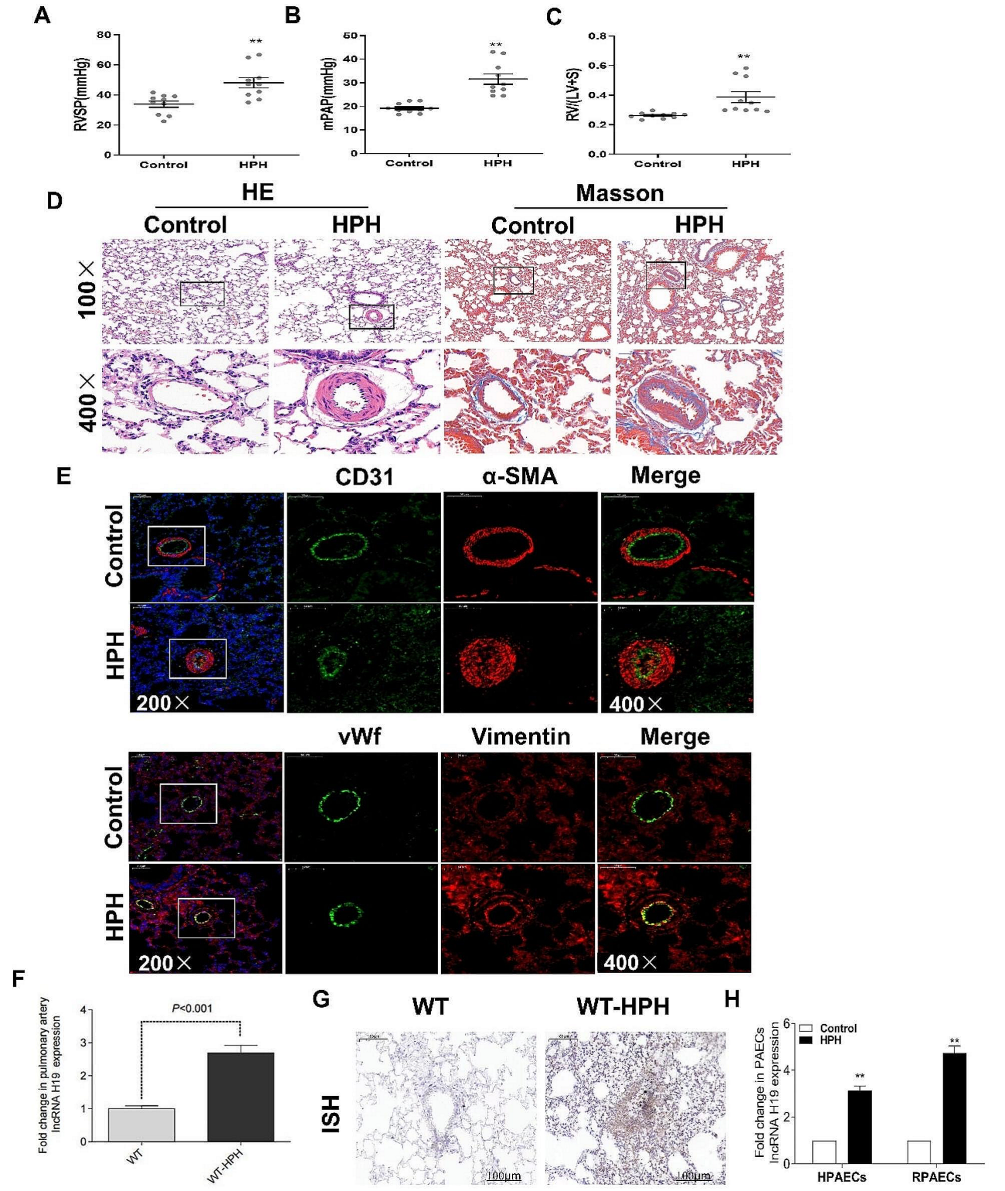
H19 was increased in HPH rats and hypoxia-treated PAECs. (**A-B**) Quantification of RVSP and mPAP in rats; (**C**) The ratio of right ventricle weight to left ventricle plus ventricular septum weight (RV/LV + S) was measured; (**D**) Morphological analysis of the pulmonary artery was performed using HE and Masson staining; (**E**) The expression of endothelial markers (CD31 or vWf) and mesothelial markers (α-SMA or vimentin) was detected using double-labelling immunofluorescence ; (**F**) The expression of H19 in pulmonary artery of rats was detected by qRT-PCR; (**G**) The expression of H19 in lung tissue of rats in each group was detected by in situ hybridization (brown)

To determine the expression patterns of H19 in the development of HPH, rats were exposed to a hypoxic environment (10% O_2_) for 28 days. In comparison to the control group, HPH rats had considerably greater RVSP and mPAP (Fig. 2A-B). Additionally, when compared with the control group, HPH rats had a far greater RV/LV + S ratio (Fig. 2C). The thickness of the pulmonary vascular wall and collagen fibrosis in the perivasculature were substantially more severe in the HPH group than in the control group, according to the results of HE and Masson staining (Fig. 2D). Previous research has demonstrated a tight connection between EndMT and pulmonary vascular remodelling in HPH patients. The expression of endothelium markers (CD31 or vWF) and mesothelial markers (α-SMA or vimentin) was detected using double-labelling immunofluorescence. As presented in Fig. 2E, the fluorescence intensity of CD31 or vWF was significantly suppressed and that of α-SMA or vimentin was increased in HPH compared with controls. Moreover, the qRT‒PCR results (Fig. 2F) showed that H19 expression was remarkably greater in the pulmonary arteries of HPH rats than it was in the control group. The in situ hybridization outcomes (Fig. 2G) showed that only a few cells in the lung tissue sections of the control group were stained with H19-specific probes. In contrast, the hypoxic group’s lung tissue had a considerably larger H19-positive region. In addition, the results of in vitro experiments showed that hypoxia (3%) treatment increased H19 levels in primary RPAECs and HPAECs (Fig. 2H).

### H19 deficiency reduced RVSP and improved RV function in HPH rats

**Fig. 3:**
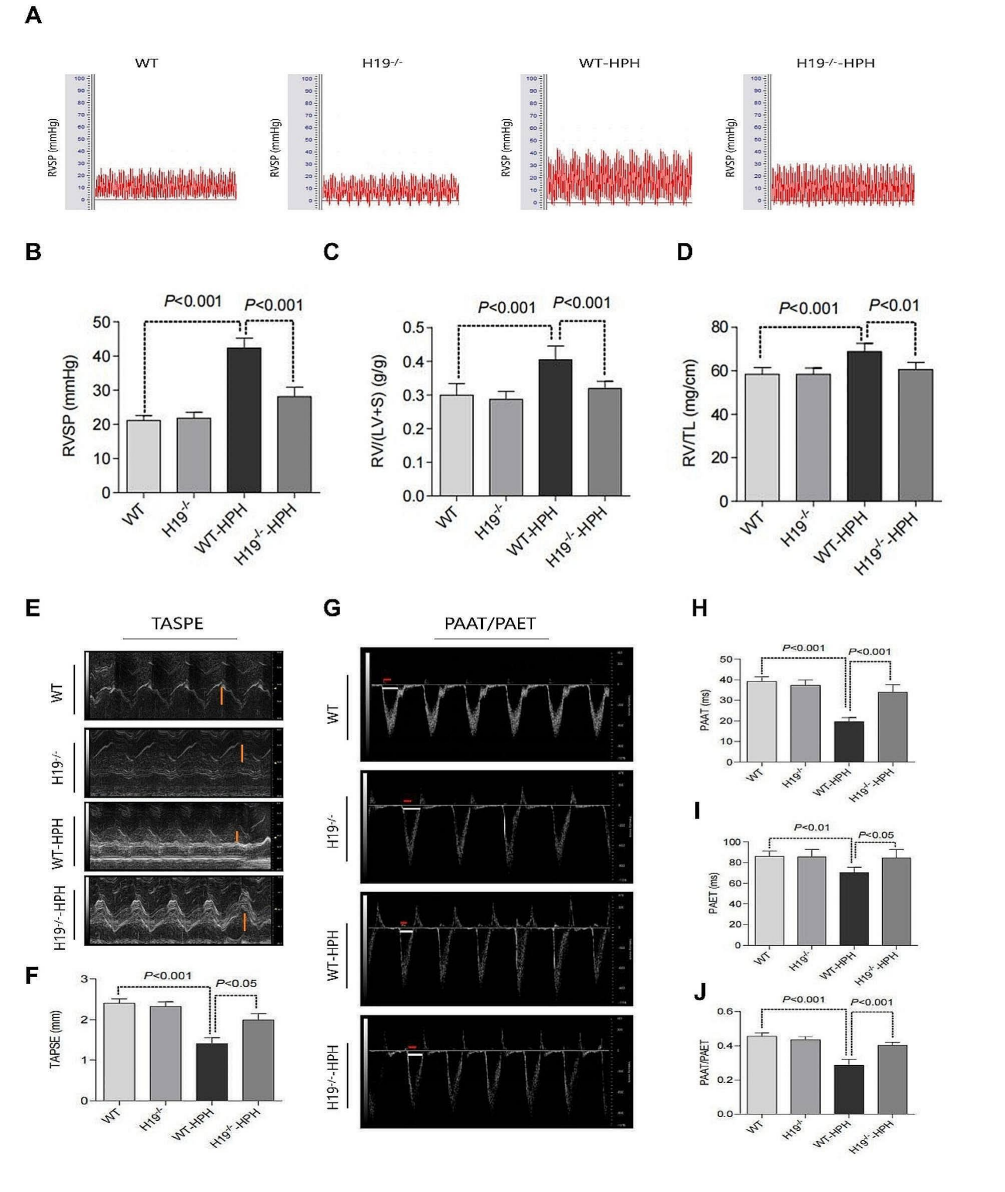
H19 deficiency reduced RVSP and improved RV function in HPH rats. (**A**) Oscillogram of right ventricular systolic pressure in rats; (**B**) Quantification of RVSP in rats; (**C**) Quantification of the ratio of RV weight to left ventricular + ventricular septal weight (RV/LV + S) in rats; (**D**) Quantification of the ratio of RV weight to tibial length (RV/TL) in rats; (**E**) The systolic displacement of tricuspid valve (brown) in rats was detected by echocardiograms in M model; (**F**) Quantification of tricuspid annular plane systolic excursion; (**G**) pulmonary artery blood flow acceleration time (red) and pulmonary artery ejection time (white) were measured by echocardiograms in Doppler model; (**H**) Quantification of pulmonary artery blood flow acceleration time; (**I**) Quantification of pulmonary artery blood flow ejection time; (**J**) Quantification of the ratio of acceleration time to ejection time. Data were presented as mean ± SEM (*n* = 6)

To further elucidate the relationship between H19 and HPH, we subjected H19-deficient rats (H19^−/−^) to hypoxia for 28 days. The genomic region of the rat H19 locus is diagrammed in Fig. S1(A). PCR Fig. S1(B) and in situ hybridization Fig. S1(C) for H19 were confirmed that H19 was successfully knocked out in SD rats. The RVSP (Fig. 3A-B), the ratio of RV/WT (Fig. 3C), RV/LV + S (Fig. 3D) and RV/TL (Fig. 3E) ratios of H19^−/−^-HPH rats considerably lower than those of the WT-HPH group. Furthermore, the WT-HPH rat exhibited a dramatic increase in TAPSE, PAAT and PAAT/PAET when compared with the control group, according to the results of echocardiographic Doppler and M-mode tracing (Fig. 3F-K). In contrast, the indices of RV function in the H19^−/−^-HPH group were similar to those in the control group. These results suggest that H19 gene deficiency can protect rats against hypoxia-induced pulmonary hypertension and RV dysfunction.

### H19 deficiency improved pulmonary vascular remodelling in HPH rats

**Fig. 4:**
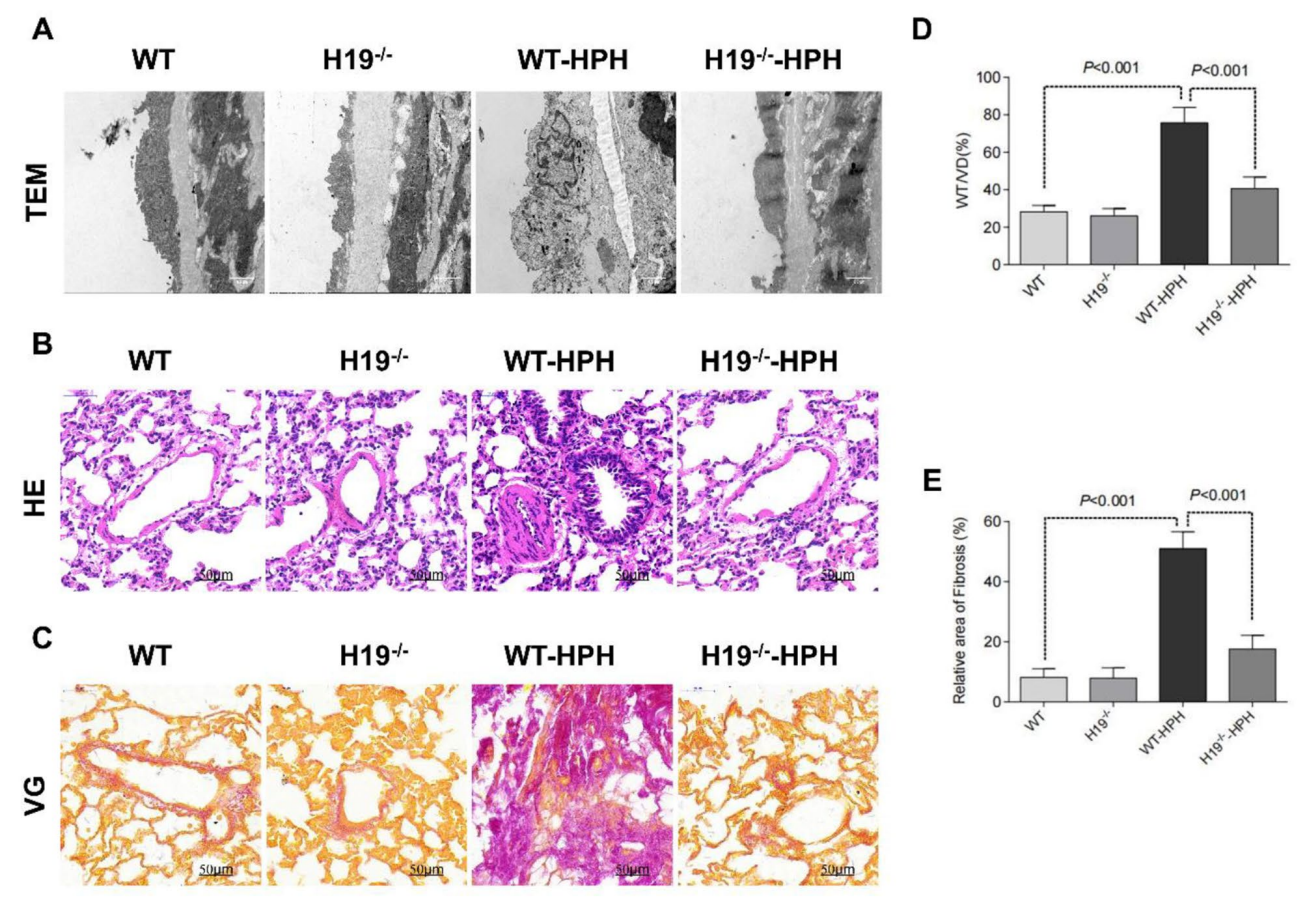
H19 deficiency improved pulmonary vascular remodelling in HPH rats. (**A**) Transmission electron microscopy (TEM) images of lung tissues; (**B**) HE staining in lung tissue; (**C**) VG staining in lung tissue; (**D**) Quantification of the ratio of pulmonary artery wall thickness to vessel diameter in rats; (**E**) Quantification of the fibrosis area

Vascular lumen stenosis caused by pulmonary artery remodelling is the root cause of increased pulmonary artery pressure and right heart failure in patients with HPH. As shown in Fig. 4A, pulmonary arteries obtained from WT-HPH group rats exhibited a ruptured elastic membrane, which was remarkably reversed by H19 deficiency. The thickness of the pulmonary vascular wall and collagen fibrosis in the perivasculature were far higher in the HPH group than in the control group, and both characteristics were obviously diminished by H19 absence, according to the results of HE and VG staining (Fig. 4B-C) The results of histomorphometric analysis further confirmed this finding (Fig. 4D-E). In conclusion, these results suggest that H19 gene knockout can improve pulmonary vascular remodelling induced by HPH in rats.

#### H19 deficiency inhibited EndMT in HPH

**Fig. 5:**
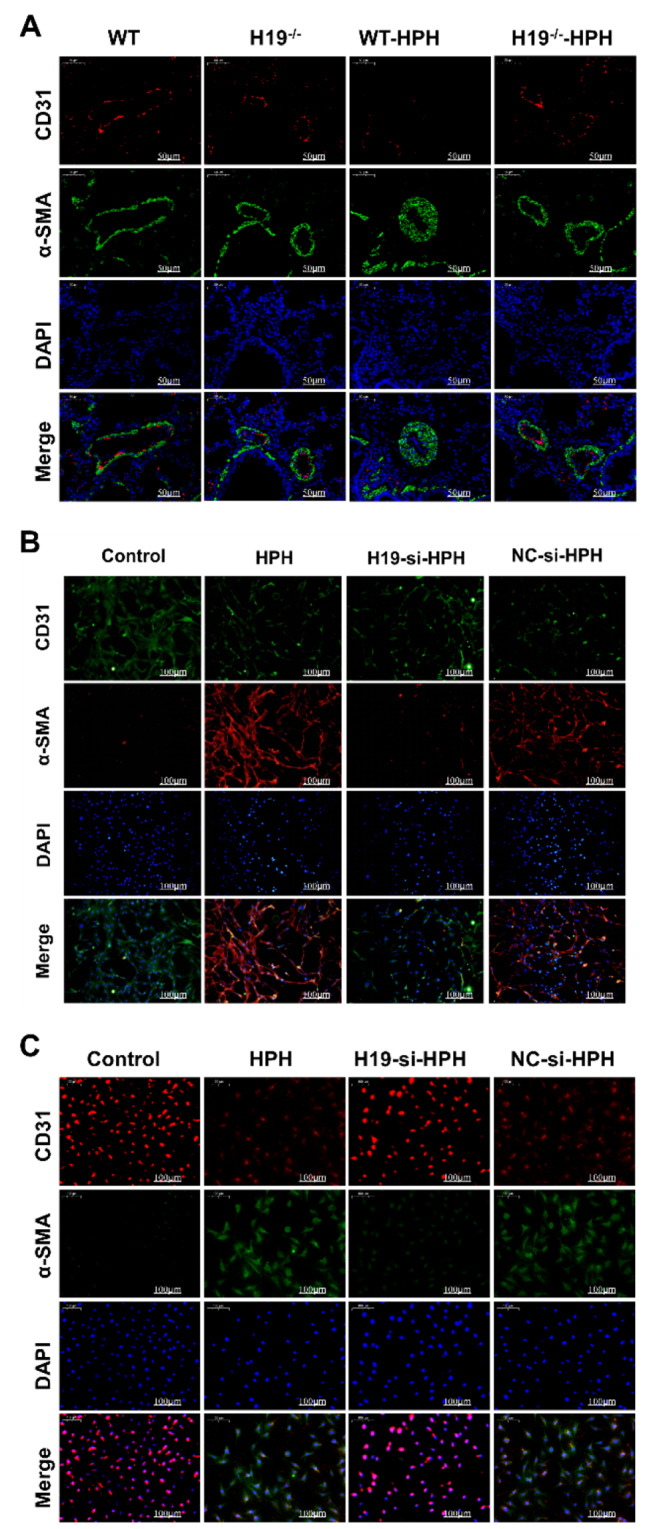
H19 deficiency inhibited EndMT in HPH. (**A**) Immunofluorescence double staining of CD31 and α-SMA in lung tissues (CD31 (red), α-SMA (green), DAPI (blue)); (**B**) Immunofluorescence double staining of CD31 and α-SMA in RPAECs; (**C**) Immunofluorescence double staining of CD31 and α-SMA in HPAECs; Data were presented as mean ± SEM (*n* = 6)

Using double-labelling immunofluorescence to identify the expression of CD31 and -SMA, the impact of H19 on EndMT in HPH rats was investigated. As presented in Fig. 5A, the fluorescence intensity of CD31 was significantly suppressed and that of α-SMA was increased in WT-HPH cells compared with controls. However, there was no discernible difference between the H19-/-HPH group rats and the control group in the CD31 and -SMA fluorescence intensities. Furthermore, we also investigated the role of H19 in EndMT in primary RPAECs (Cell identification results were shown in Fig. S2) and HPAECs and found that H19 knockdown significantly reduced hypoxia-induced EndMT in both cells. (Fig. 5B-C). These results suggest that H19 deficiency-improved pulmonary artery remodelling is associated with inhibiting EndMT.

### H19 deficiency regulated EndMT of PAECs via the let-7 g/TGFβR1 axis

**Fig. 6:**
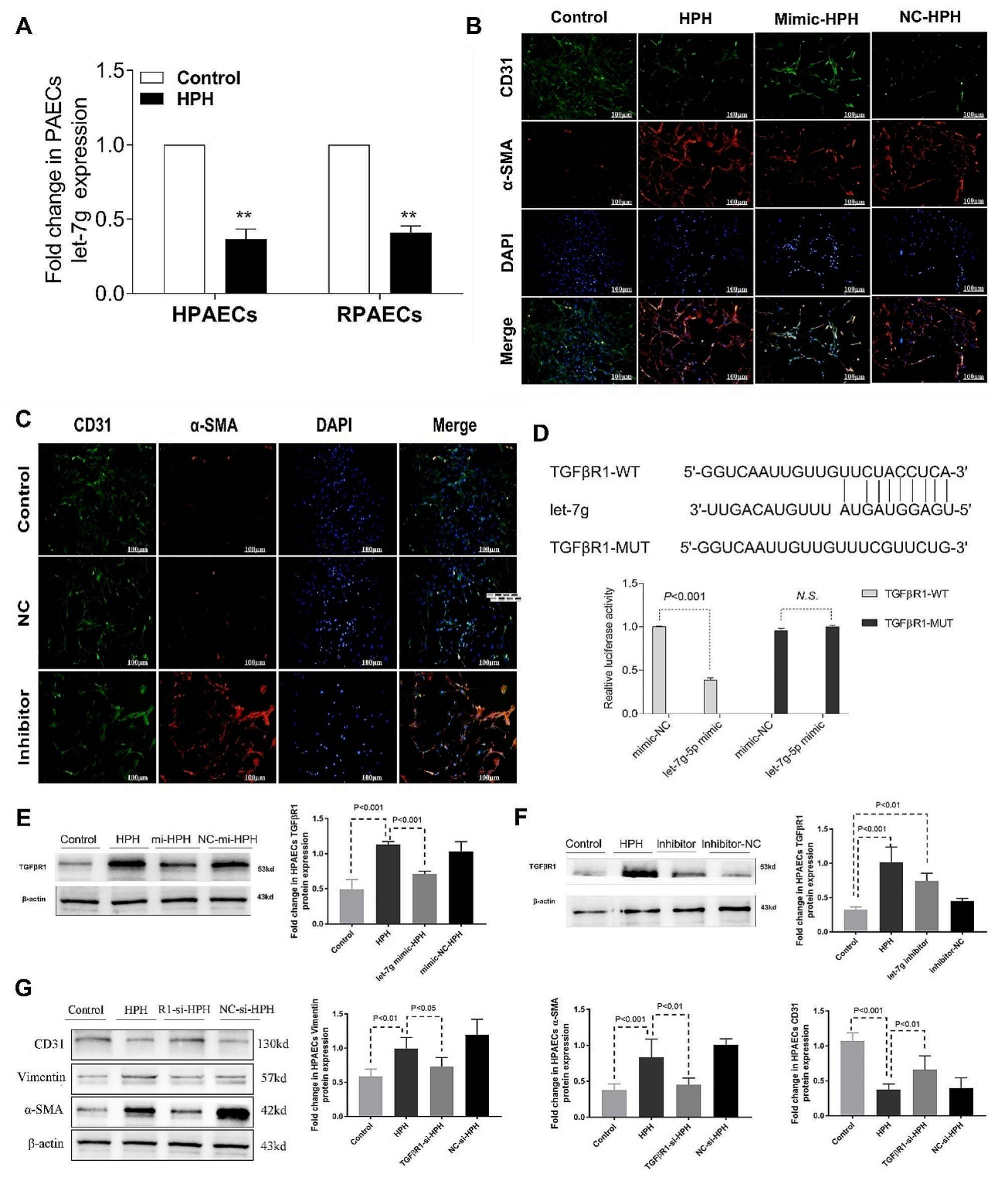
H19 deficiency regulated EndMT of PAECs via the let-7 g/TGFβ signalling. (**A**) The expression level of let-7 g in PAECs ;(**B**) Immunofluorescence double staining of CD31 and α-SMA in RPAECs treated with let-7 g mimic; (**C**) Immunofluorescence double staining of CD31 and α-SMA in RPAECs treated with let-7 g inhibitor; (**D**) the potential binding sites of let-7 g to TGFβR1 predicted by Targetscan 7.0 and quantification of the fluorescence intensity in HEK293T cells; (**E-F**) After transfection of let-7 g mimic or inhibitor, the expression level of TGFβR1 protein in HPAECs; (**G**) Transfection of after transfection of TGFβR1-Si, the expression levels of CD31, Vimentin and α-SMA were detected by Western blot

Let-7 g was previously discovered to be downregulated in the pulmonary arteries of HPH rats and inhibited hypoxia-induced proliferation of PASMCs [7, 23]. Interestingly, we subsequently found through bioinformatics that let-7 g may also be closely related to EndMT at PH [24]. In this study, we discovered that PAECs exposed to hypoxia displayed a clearly decreased expression of let-7 g (Fig. 6A). The let-7 g mimic significantly inhibited EndMT in RPAECs (Fig. 6B) and HPAECs (Fig.S3), but the let-7 g inhibitor directly stimulated EndMT (Fig. 6C). In terms of mechanism, TGFβR1 is a key protein in the TGFβ/Smad signalling pathway and plays an important role in EndMT. By using a luciferase reporter assay (Fig. 6D), we were able to further confirm that let-7 g may bind to the TGF-R1 3′UTR and negatively control the production of TGF-R1 in PAECs (Fig. 6E-F). In addition, knockdown of endogenous TGFβR1 with TGFβR1-siRNA efficaciously decreased hypoxia-induced Vimentin and α-SMA expression in HPAECs and restored CD31 protein levels (Fig. 6G). These results demonstrated that let-7 g/TGFβ signalling is important for EndMT in HPH.

**Fig. 7:**
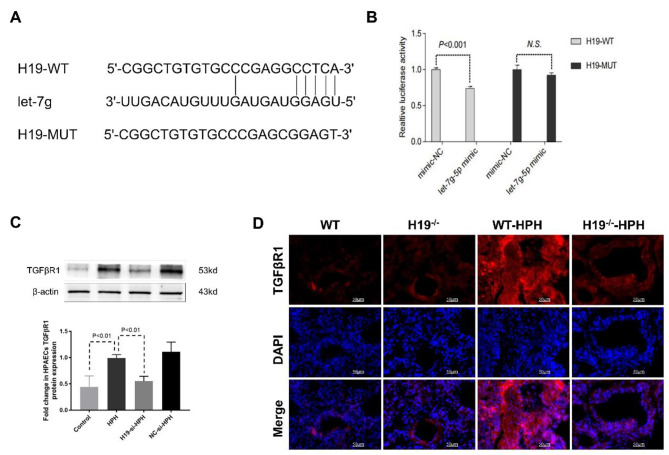
H19 deficiency ameliorated EndMT was associated with miR-let-7 g/TGFβ signalling. (**A**) The potential binding targets of H19 to let-7 g was predicted by RegRNA 2.0 software; (**B**) Quantification of the fluorescence intensity in HEK293T cells; (**C**) After transfection with H19-Si, the expression level of TGFβR1 protein in HPAECs was detected by Western blot; (**D**) Immunofluorescence staining of TGFβR1 in lung tissues (TGFβR1 (red), DAPI (blue)). Data were presented as mean ± SEM (*n* = 6)

According to recent research, lncRNAs can act as naturally occurring competitive RNAs (ceRNAs) to scavenge miRNAs that bind to the 3′UTR of mRNAs for their degradation. We found that H19 could target binding with let-7 g by bioinformatics websites (RegRNA 2.0) (Fig. 7A) and verified by the luciferase reporter assay (Fig. 7B). In addition, we found that H19 deficiency led to a considerable decrease in the amount of TGF-R1 expression that is elevated by hypoxia in both HPAECs (Fig. 7C) and lung tissue (Fig. 7D). These results demonstrated that H19 may regulate EndMT in PAECs through the let-7 g/TGFβR1 signalling axis.

## Discussion

A gradual rise in pulmonary artery pressure and thickening of the pulmonary artery wall are two features of the complicated cardiovascular disease HPH [25]. Several lncRNAs have been shown to have a role in HPH pathogenesis in recent years [26, 27]. In this study, we discovered that H19 was both increased by hypoxia in vitro and abundantly expressed in HPH rodent lungs. Moreover, H19 deficiency in vivo can effectively prevent pulmonary artery remodelling and right heart failure induced by hypoxia. Mechanistically, we found that TGFβR1 is a novel target of let-7 g and that H19 upregulated TGFβR1 expression by sponging let-7 g following hypoxia stimulation. Moreover, both in vivo and in vitro, H19 absence prevented EndMT in HPH cells.

Vascular remodelling in PH is caused by excessive pulmonary artery smooth muscle cell proliferation and abnormal arterial endothelial cells [28]. A previous study revealed that H19 was highly expressed in monocrotaline-induced PAH and promoted the proliferation of PASMCs [29]. EndMT, a phenotypic change in endothelial cells, is increasingly becoming recognized as the crucial cytopathological basis of HPH in research [30, 31]. Under hypoxic stimulation, PAECs can lose their original structural characteristics and phenotypes and then transdifferentiate into smooth muscle-like cells, which have high proliferative activity and contribute to the remodelling process of the pulmonary artery [11, 32]. Our study investigated the role of H19 in HPH for the first time and found that H19 plays an important role during the conversion of PAECs into smooth muscle-like cells of under HPH. In the current experiment, we found that hypoxia treatment for 4 weeks can significantly promote pulmonary artery remodelling and EndMT in rats, and H19 knockout can significantly improve pathological changes. As a result, we propose that H19 may be a key player in the proliferation of PASMCs, EndMT, and right myocardial hypertrophy, making it an appropriate therapeutic target for PH. However, a recent study indicated that knockdown of H19 significantly promoted the proliferation of rPASMCs, which seems to contradict our finding. Different blood samples, different models, tissues and cell samples may have caused some discrepancies [33]. 

H19 with a primary structure up to 2.3 KB can act as a ceRNA to adsorb miRNA, thereby weakening its interference with the targeted mRNA [29, 34]. Our findings imply that H19 sponges let-7 g, which is consistent with a prior publication [35]. Let-7 g is a member of the let-7 family, whose role in angiogenesis, vascular remodelling and epithelial mesenchymal transformation has been well documented [36, 37]. In addition, let-7 g was found to be downregulated in remodelled pulmonary arteries of HPH rats, whereas restoring let-7 g could markedly blunt hypoxia-induced cell proliferation in PASMCs [7, 23]. We subsequently found through bioinformatics that let-7 g may also be closely related to EndMT at PH [24] and verified in this study. Other members of the Let-7 family, besides let-7 g, also take part in PH. Let-7b inhibited PASMC growth in monocrotaline-induced PAH, according to a prior study [29]. Moreover, the increased expression of ET-1 caused by the decreased let-7b expression influenced the proliferation of PASMC and PAEC in chronic thromboembolic PH [38]. It is well established that the TGF signalling pathway is essential for the development of HPH and vascular remodeling [39, 40]. Our results were the first to confirm that TGFβR1, a key protein in the TGFβ signalling pathway, is a new target of let-7 g and that the H19-let-7 g-TGFβR1 axis, which stimulates EndMT, plays a role in the pathophysiology of HPH.

This study does have certain restrictions, though. First off, while discovering by DEG analysis that H19 expression increased in the lung tissue of PH patients, we did not investigate H19 expression in the serum and lung tissue of HPH patients. Could blood H19 be used to diagnose or predict outcomes in PH patients? Second, it is not quite obvious how precisely hypoxia controls H19. H19 was strongly expressed in the 1-day-old rat aorta but was not found in the adult aorta, as was previously reported. H19 transcripts were hardly present in the carotid artery before injury, but they were plentiful at 7 and 14 days later and were largely localized to the neointima by in situ hybridization [41]. Furthermore, hypoxia has been shown to promote carcinogenic effects in glioblastoma by directly and indirectly inducing H19 expression through hypoxia-inducible transcription factor 1 alpha (HIF-1α ) activity [42]. There is a functional link between HIF-1α and H19 that determines H19 elevation in hypoxic cancer cells [43]. HIF-1, one of the hypoxia-responsive factors, is the main regulator of oxygen homeostasis and hypoxic adaptation in the lung. PASMC growth, EndMT development, and pulmonary vascular remodelling are all influenced by aberrant HIF-1 activation during PH etiology [44, 45]. Consequently, we propose that the overexpression of H19 may be caused by both abnormal HIF-1 activity and injured pulmonary small arteries in PH. But more evidence is required to support the hypothesis.

**Fig. 8:**
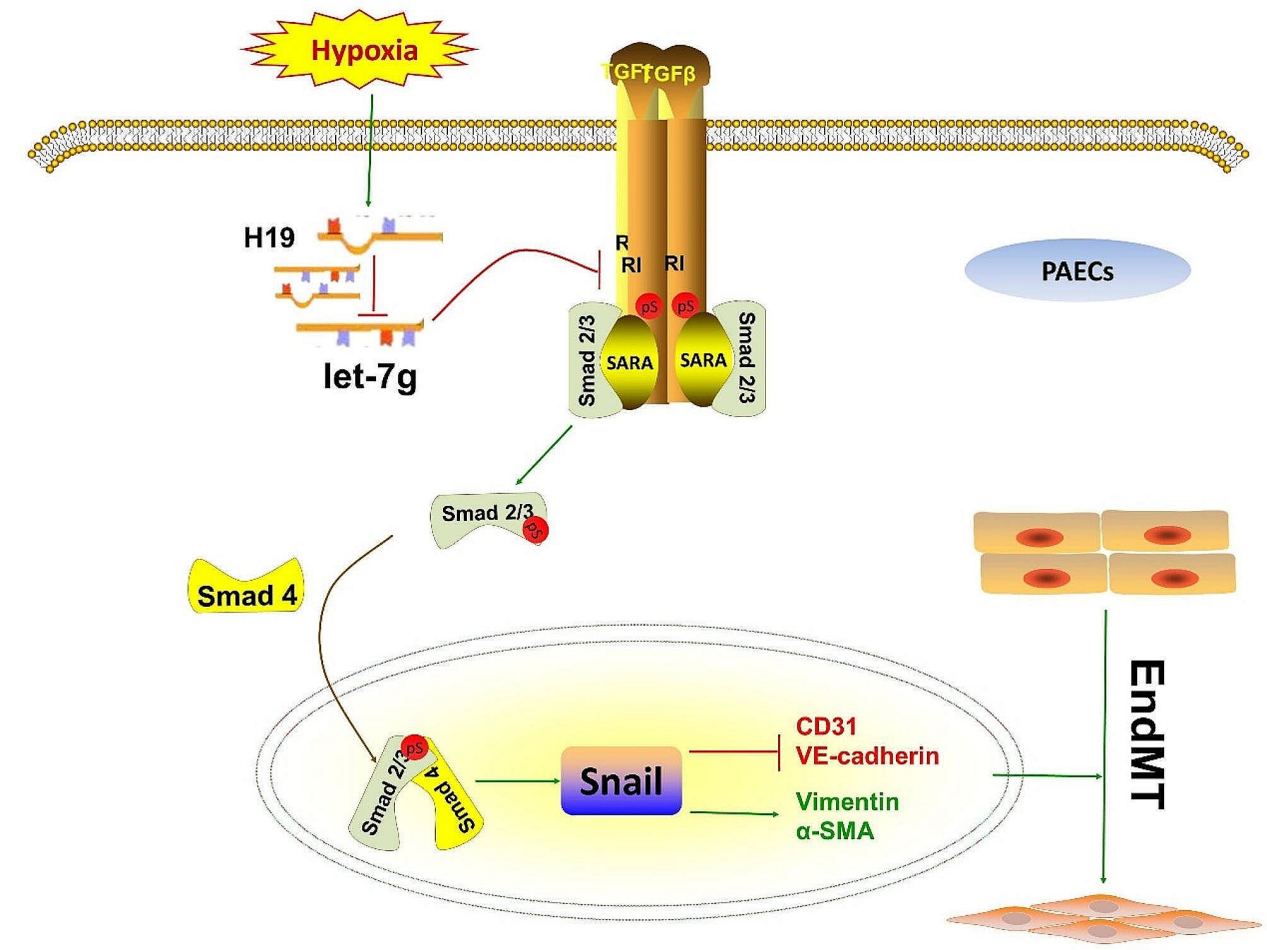
Scheme of the regulation of H19-let-7 g-TGFβR1 axis to EndMT in HPH

In summary, this work demonstrates that the expression of H19 was increased in the pulmonary arteries of HPH rats and hypoxic PAECs. In HPH rats, H19 absence alleviated right ventricular dysfunction and pulmonary vascular remodelling, which were both related to EndMT suppression via let-7 g/TGFβ signalling (Fig. 8). Our data imply that H19 could provide a novel treatment target for HPH.

### Electronic supplementary material

Below is the link to the electronic supplementary material.

Supplementary Material 1

Supplementary Material 2

Supplementary Material 3

Supplementary Material 4

## Data Availability

No datasets were generated or analysed during the current study.

## Abbreviations

EndMT: Endothelial-to-mesenchymal transition
HPH: Hypoxia-induced pulmonary hypertension
PAECs: Pulmonary artery endothelial cells
PASMCs: Pulmonary artery smooth muscle cells
