# Supplementary material for: LncRNAH19 acts as a ceRNA of let-7 g to facilitate endothelial-to-mesenchymal transition in hypoxic pulmonary hypertension via regulating TGF-β signalling pathway
Source: Respir Res. 2024 Jul 10;25:270. doi: 10.1186/s12931-024-02895-y (PMC11238495; doi:10.1186/s12931-024-02895-y)
Supplement: Supplementary file 1 — Supplementary Material 1 [file 12931_2024_2895_MOESM1_ESM.docx]

**
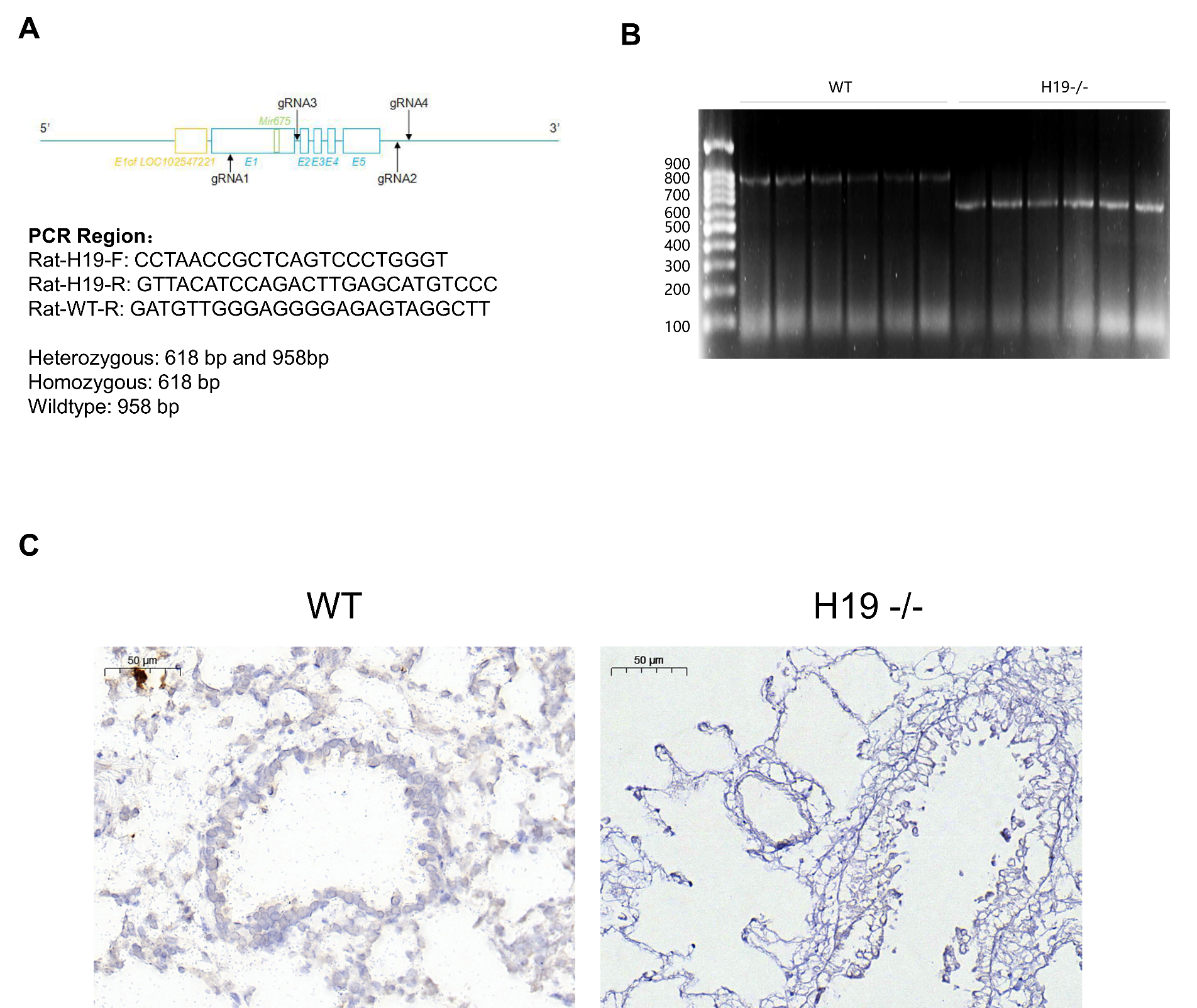
**

**Figure** **Supplemental 1 H19 knockout rats were confirmed by genotyping and in situ hybridization**

1. Genomic region of rat H19 locus and create a H19 knockout rat model (SD) by CRISPR/Cas-mediated genome engineering (gene is oriented from left to right, total size is 2.68 kb). Solid bars represent ORF, open bars present UTRs. (B) The expression of H19 in lung tissue of rats in each group was detected by in situ hybridization (brown). (C) The genotypes of the H19 gene of rats were determined by PCR.
