# Supplementary material for: LncRNAH19 acts as a ceRNA of let-7 g to facilitate endothelial-to-mesenchymal transition in hypoxic pulmonary hypertension via regulating TGF-β signalling pathway
Source: Respir Res. 2024 Jul 10;25:270. doi: 10.1186/s12931-024-02895-y (PMC11238495; doi:10.1186/s12931-024-02895-y)
Supplement: Supplementary file 2 — Supplementary Material 2 [file 12931_2024_2895_MOESM2_ESM.docx]

**
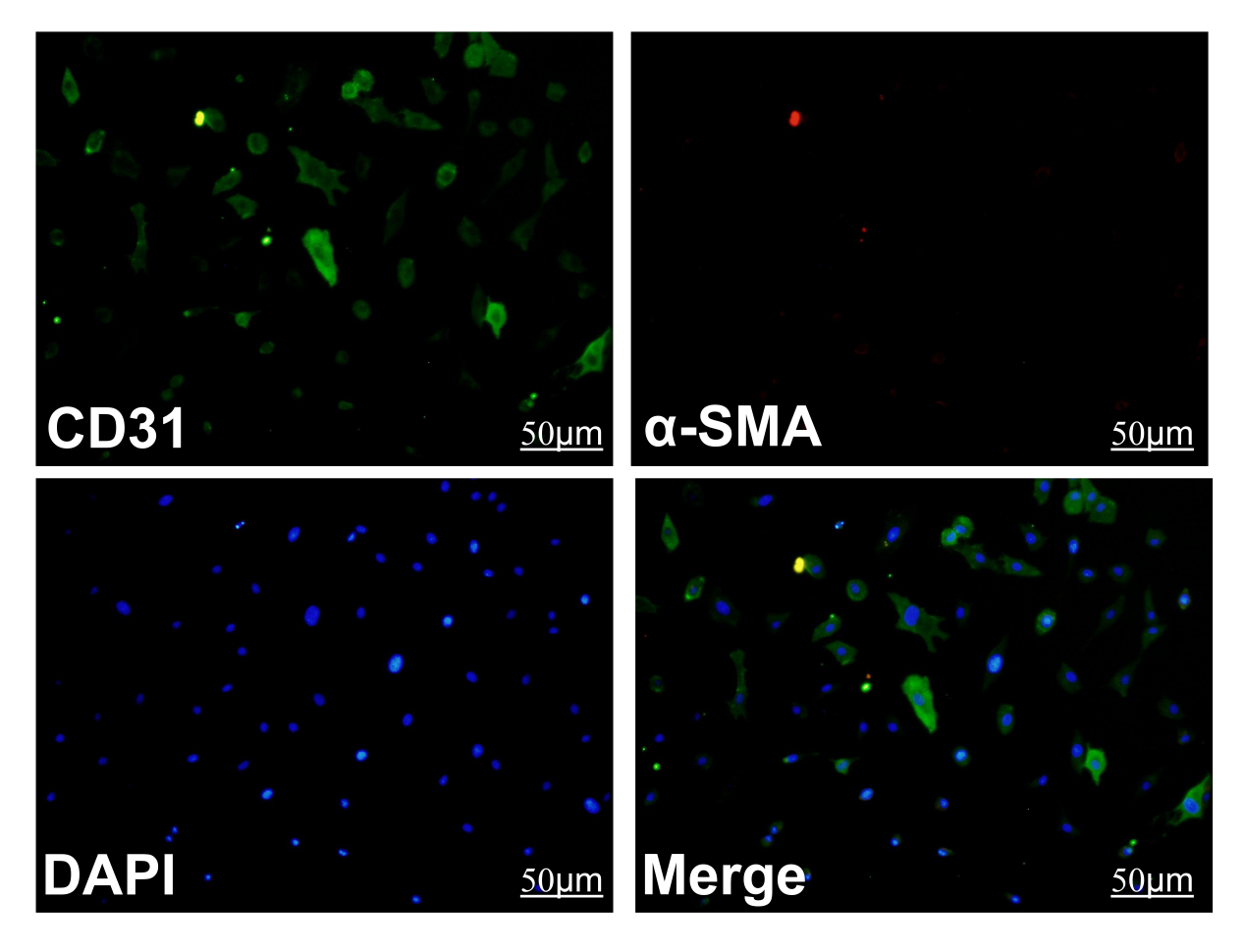
**

**Figure Supplemental 2 Primary RPAECs identification by indirect immunofluorescence for CD31 and α-SMA.**
