# Supplementary material for: LncRNAH19 acts as a ceRNA of let-7 g to facilitate endothelial-to-mesenchymal transition in hypoxic pulmonary hypertension via regulating TGF-β signalling pathway
Source: Respir Res. 2024 Jul 10;25:270. doi: 10.1186/s12931-024-02895-y (PMC11238495; doi:10.1186/s12931-024-02895-y)
Supplement: Supplementary file 3 — Supplementary Material 3 [file 12931_2024_2895_MOESM3_ESM.docx]

**
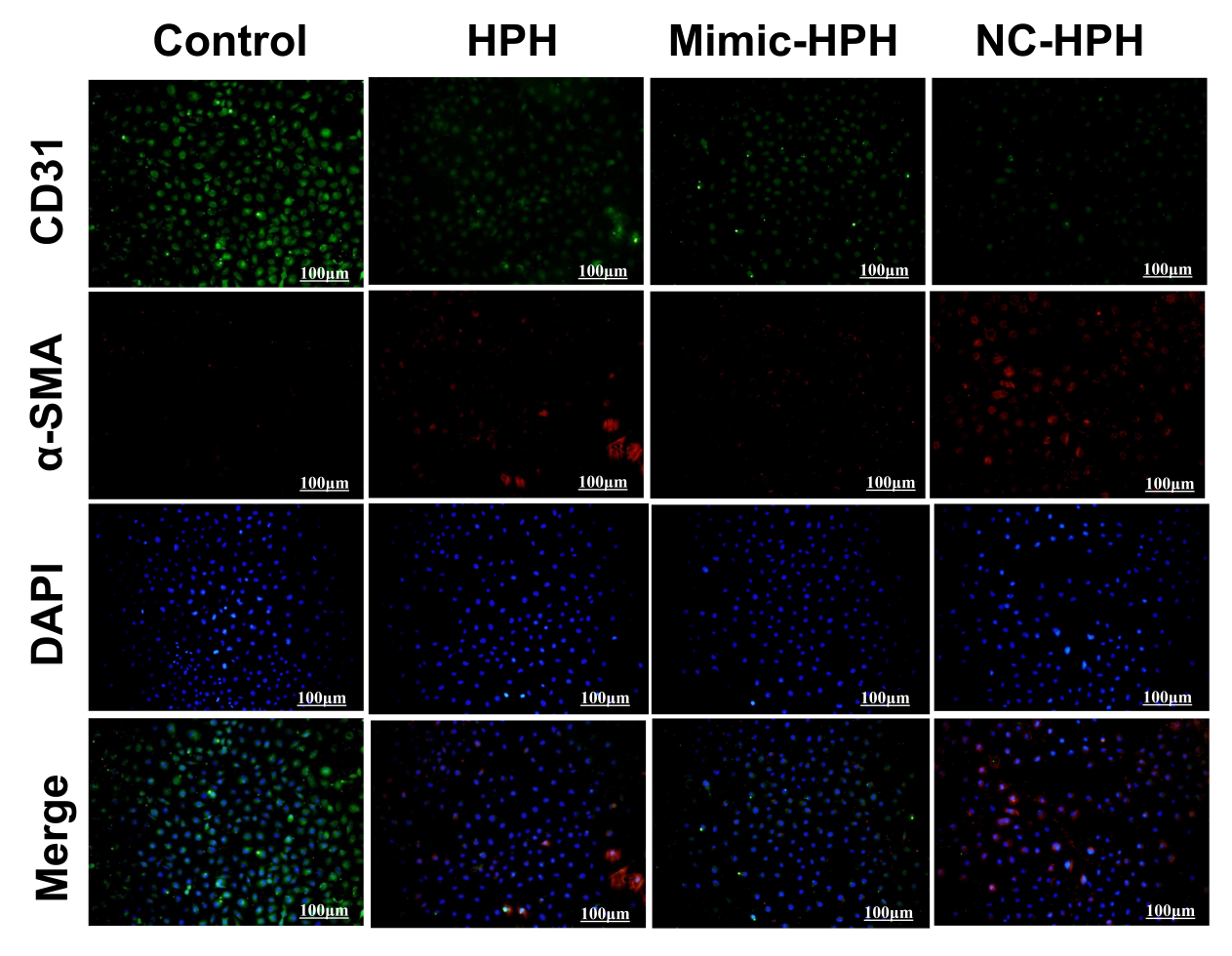
**

**Figure Supplemental 3 Immunofluorescence double staining of CD31 and α-SMA in HPAECs treated with let-7g mimic.**
