# Supplementary material for: LncRNAH19 acts as a ceRNA of let-7 g to facilitate endothelial-to-mesenchymal transition in hypoxic pulmonary hypertension via regulating TGF-β signalling pathway
Source: Respir Res. 2024 Jul 10;25:270. doi: 10.1186/s12931-024-02895-y (PMC11238495; doi:10.1186/s12931-024-02895-y)

**Original image for checking**

**Figure 6E**

TGFβR1 (53kd)


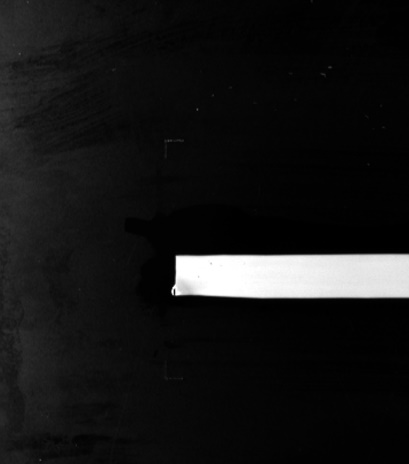

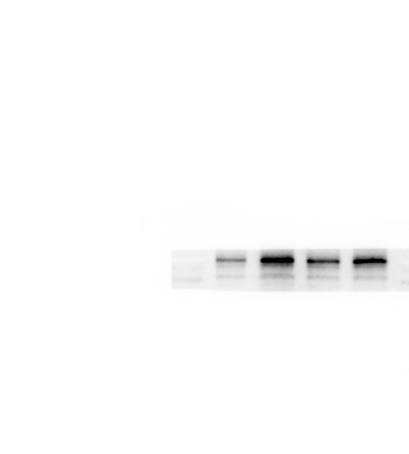


β-actin (43kd)


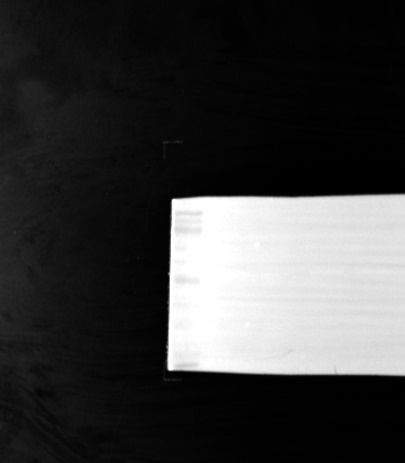

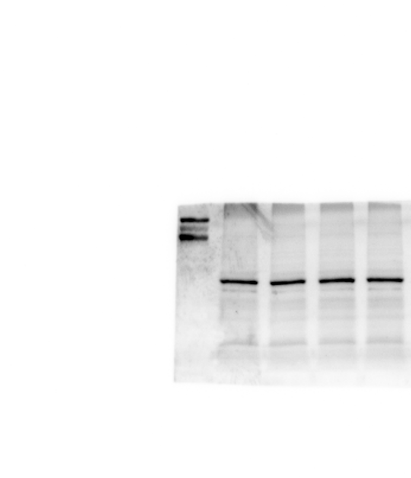


**Figure 6F**

TGFβR1 (53kd)


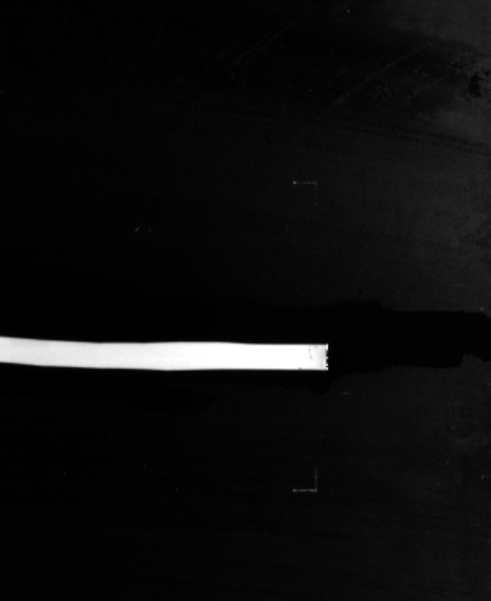

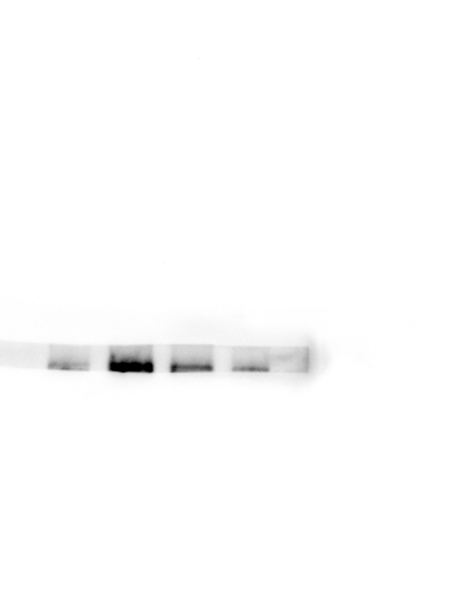


β-actin (43kd)


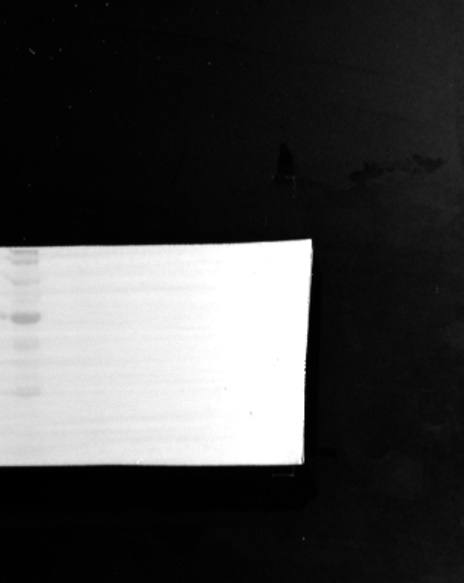

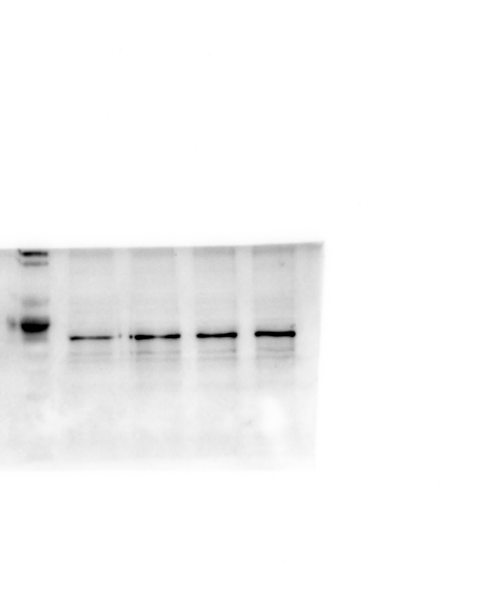


**Figure 6G**

α-SMA (42kd)


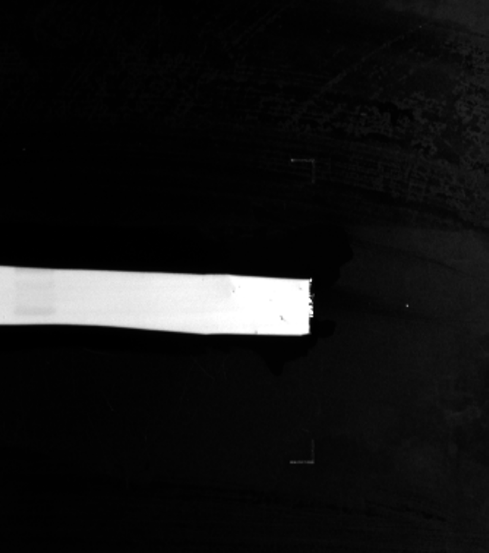

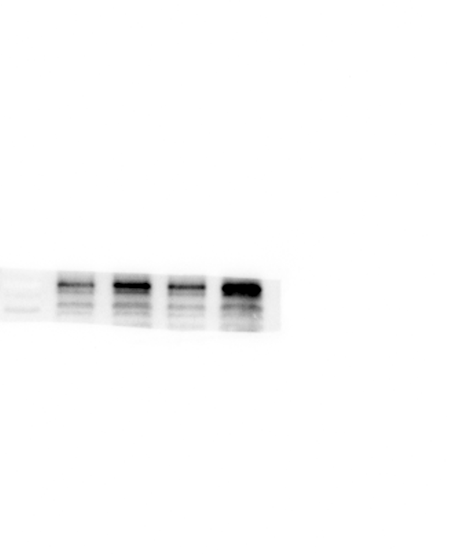


CD31 (130kd)


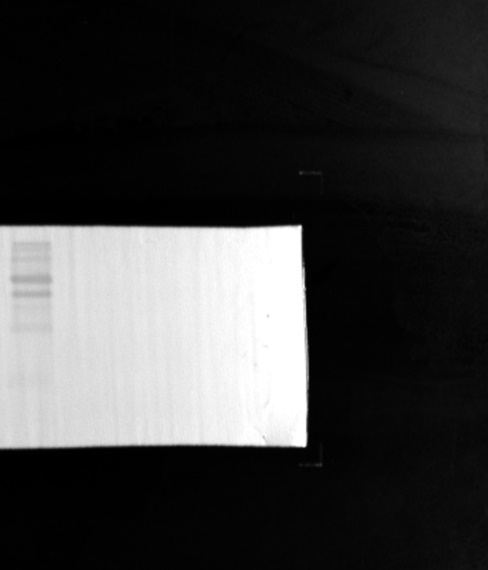

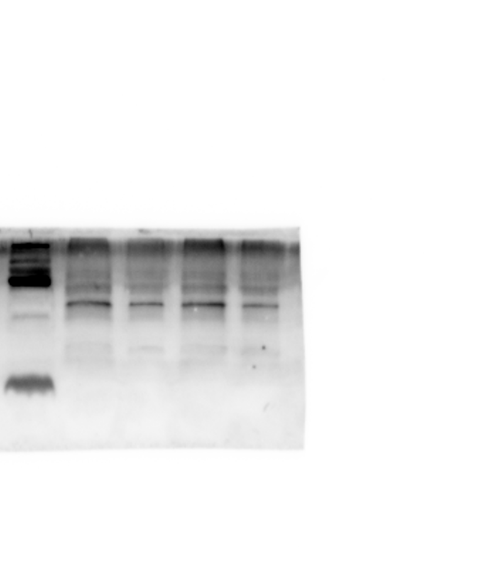


Vimentin (57kd)


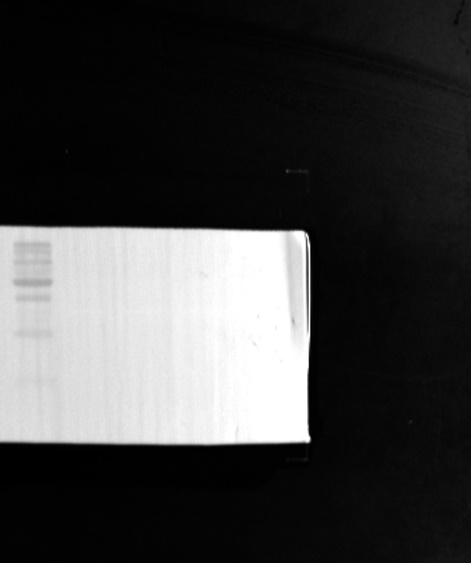

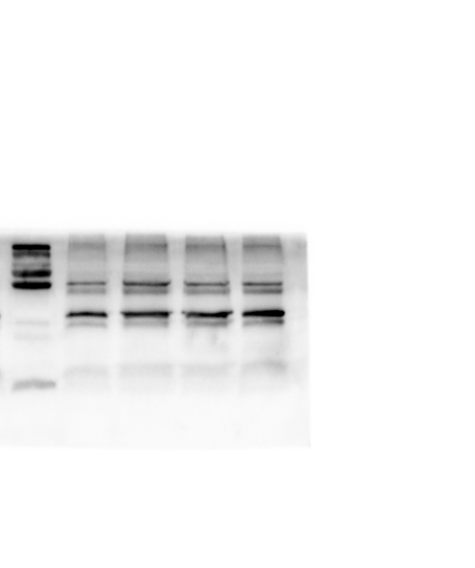


β-actin (43kd)


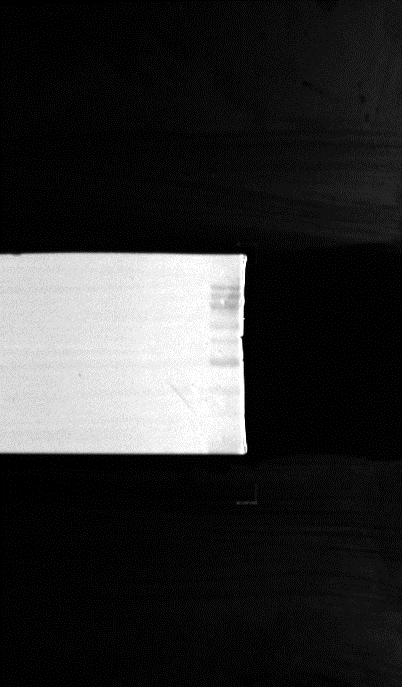

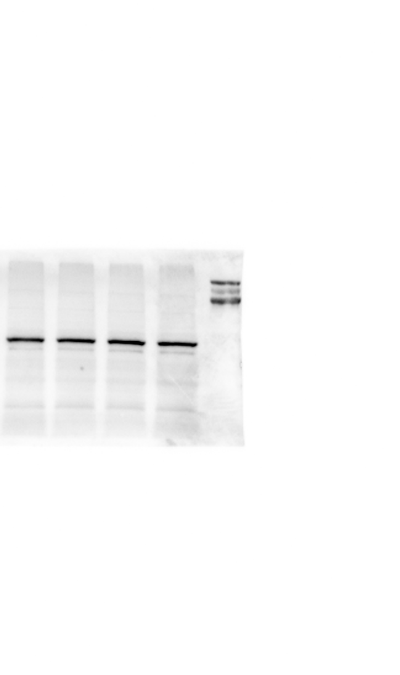


**Figure 7C**

TGFβR1 (53kd)


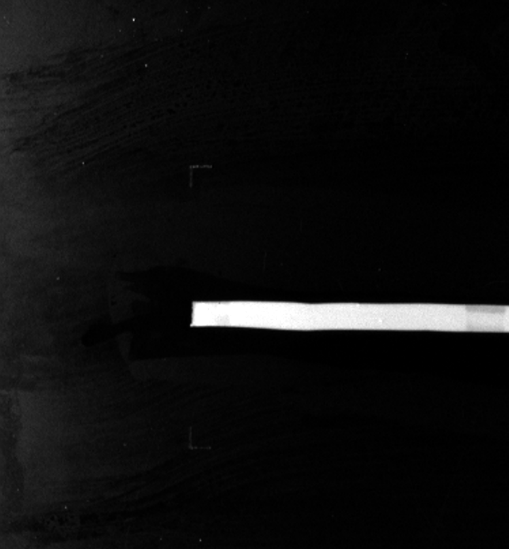

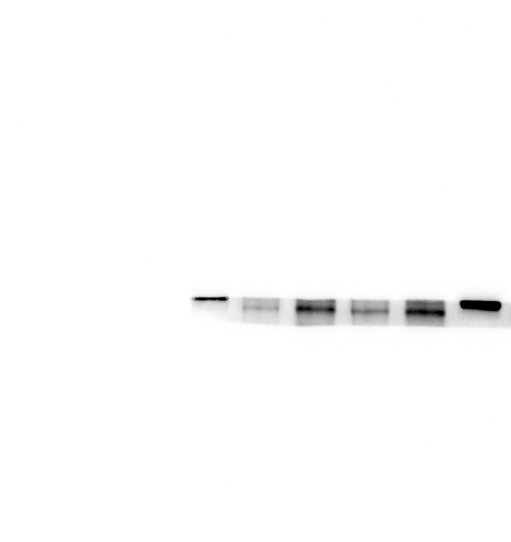


β-actin (43kd)


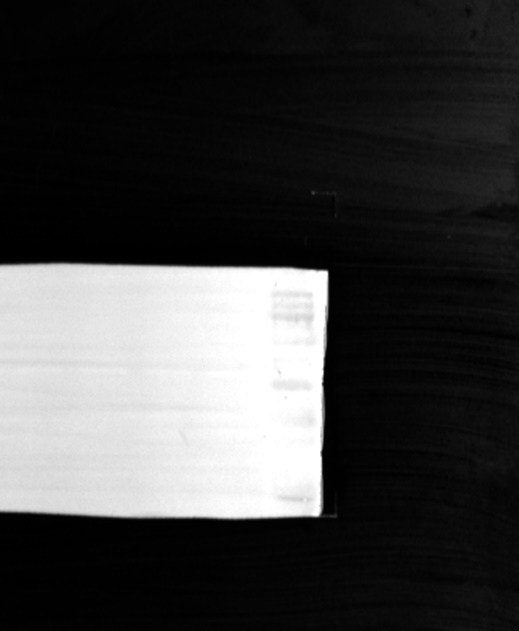

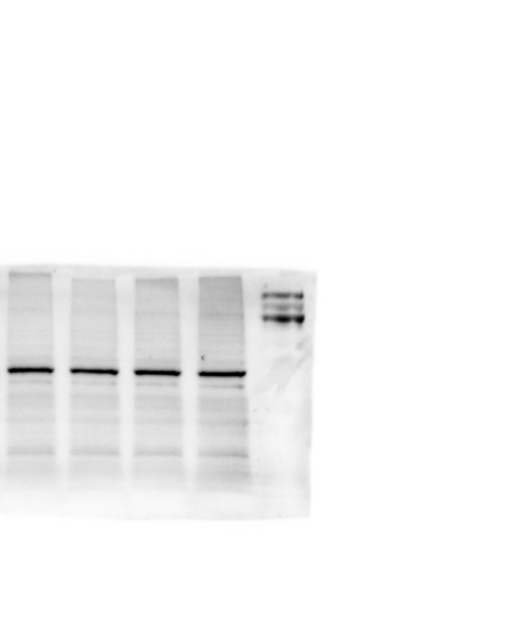

Supplement: Supplementary file 4 — Supplementary Material 4 [file 12931_2024_2895_MOESM4_ESM.docx]
